# Strontium ranelate reduces the risk of vertebral fracture in young postmenopausal women with severe osteoporosis

**DOI:** 10.1136/ard.2008.094516

**Published:** 2008-08-18

**Authors:** C Roux, J Fechtenbaum, S Kolta, G Isaia, J B Cannata Andia, J-P Devogelaer

**Affiliations:** 1 Département de Rhumatologie, AP-HP Hôpital Cochin, Université Paris-Descartes, Paris, France; 2 Divisione Universitaria di Medicina Generale, Torino, Italy; 3 Servicio de Metabolismo Oseo y Mineral, Instituto Reina Sofia de Investigacion, Hospital Universitario Central de Asturias, Oviedo, Spain; 4 Arthritis Unit, Université Catholique de Louvain, Brussels, Belgium

## Abstract

**Objectives:**

Early osteoporotic fractures have a great impact on disease progression, the first fracture being a major risk factor for further fractures. Strontium ranelate efficacy against vertebral fractures is presently assessed in a subset of women aged 50–65 years.

**Methods:**

The Spinal Osteoporosis Therapeutic Intervention (SOTI) was an international, double blind, placebo controlled trial, supporting the efficacy of strontium ranelate 2 g/day in reducing the risk of vertebral fractures in postmenopausal women with osteoporosis and a prevalent vertebral fracture. 353 of these randomly assigned women were included in this analysis.

**Results:**

Over 4 years, strontium ranelate significantly reduced the risk of vertebral fracture by 35% (relative risk 0.65; 95% CI 0.42 to 0.99, p<0.05). In the strontium ranelate group, the bone mineral density increased from baseline by 15.8% at lumbar spine and 7.1% at femoral neck.

**Conclusion:**

These data demonstrate a significant vertebral antifracture efficacy of strontium ranelate in young postmenopausal women aged 50–65 years with severe osteoporosis and confirm the efficacy of this antiosteoporotic treatment to prevent vertebral fractures, whatever the age of the patient.

Vertebral fractures represent 27% of all osteoporotic fractures coming to clinical attention^1^ and their deleterious consequences on health are now well recognised. Clinical vertebral fractures increase mortality in the elderly.^2^ ^3^ Prevalent vertebral fractures increase the risk of subsequent vertebral and non-vertebral fractures,^3^ ^4^ as well as the risk of hip fracture with at least a twofold excess. The risk of further fracturing has been shown to be higher among younger people compared with the elderly.^4^ Few data are available in clinical trials in patients younger than 65 years.

Strontium ranelate is an antiosteoporotic treatment that decreases the risk of vertebral^5^ and non-vertebral fractures,^6^ including the risk of hip fractures in a high-risk population.^6^ The aim of the present study was to assess the efficacy of strontium ranelate in patients with osteoporosis aged 50–65 years, most presenting with a prevalent vertebral fracture, a subgroup of patients having a very high lifetime risk of fractures.

## MATERIALS AND METHODS

### Study subjects

Data from the Spinal Osteoporosis Therapeutic Intervention (SOTI) trial were used for this study. In this randomised, double blind, placebo controlled clinical trial, 1649 postmenopausal patients aged 50 years or more were enrolled with these inclusion criteria: at least one vertebral fracture and a lumbar spine bone mineral density (BMD) of 0.840 g/cm^2^ or less. The vertebral fracture incidence was the main efficacy criterion of the study. The assessment of this criterion was performed over 3 years (main statistical analysis) showing an early and sustained significant reduction in the vertebral fracture risk by 49% over the first year and by 41% over 3 years.^5^ Furthermore, a pre-planned analysis was performed over 4 years showing a 33% reduction (relative risk (RR) 0.67; 95% CI 0.55 to 0.81, p<0.001) in the risk of sustaining a vertebral fracture over 4 years (data on file).

For this post-hoc analysis investigating, blinded to treatment, the antifracture efficacy of strontium ranelate in younger postmenopausal women with osteoporosis, we selected the subset of the SOTI study population aged 50–65 years.

### Treatment regimens

Patients were randomly assigned to receive 2 g per day of strontium ranelate or placebo. Throughout the study period, subjects received daily calcium and vitamin D supplements at lunchtime.

### Assessment of outcomes

Radiographs of the spine were obtained at baseline and annually and assessed centrally. A semiquantitative visual assessment of each vertebrae from T4 to L4 was performed by the same reader throughout the study, using the semiquantitative grading scale as previously described by Genant *et al*.^7^

BMD at the lumbar spine and proximal femur was measured by dual-energy *x* ray absorptiometry at baseline and at 6-month intervals. For patient diagnostic categorisation, lumbar spine and femoral neck BMD T-scores were calculated using a reference population previously described.^8^ Femoral neck BMD T-scores were also re-calculated using the National Health and Nutrition Examination Survey III (NHANES III) reference.

### Statistical analysis

The primary endpoint of this analysis was the incidence in patients experiencing a new vertebral fracture, estimated according to the Kaplan–Meier method, and an adjusted Cox model was used to compare groups and to estimate the RR of experiencing a new vertebral fracture and its 95% CI. Secondary endpoints included the incidence of non-vertebral fractures and spine and hip BMD changes.

The adjusted Cox model considered the treatment effects and the following covariates: country, L2/L4 BMD at baseline and prevalent vertebral fractures.

## RESULTS

Among the patients included in the SOTI study, 385 were aged 50–65 years, of whom 353 were eligible for the assessment of the efficacy of strontium ranelate on vertebral fractures according to the intention-to-treat principle (at least one sachet intake, at least one assessable vertebral *x* ray at baseline and one post-baseline). Baseline characteristics of this population are presented in table 1. There was no statistically significant difference between the patients given 2 g/day strontium ranelate (N  =  168) and those given placebo (N  =  185).

**Table 1:** Baseline characteristics of patients

	Placebo	Strontium ranelate	p Value*
	N = 185	N = 168	
Age, years	60 (3.4)	60 (3.6)	NS
Years since menopause	13.4 (6.1)	13.5 (6.0)	NS
BMI (kg/m^2^)	26.1	26.7	NS
⩾1 Prevalent vertebral fracture (%)	153 (82.7)	131 (78.0)	NS
Previous non-vertebral fracture (%)	50 (27.0)	31 (18.5)	NS
Bone mineral density			
Lumbar spine T score	−3.7 (1.0)	−3.5 (1.2)	NS
Femoral neck T score	−2.5 (0.8)	−2.5 (0.8)	
Femoral neck T score (NHANES III reference)	−1.92	−1.92	

Data are presented as mean values (SD).

*Wilcoxon test between the two groups of treatment.

BMI, body mass index; NHANES, National Health and Nutrition Examination Survey III.

**Figure 1:**
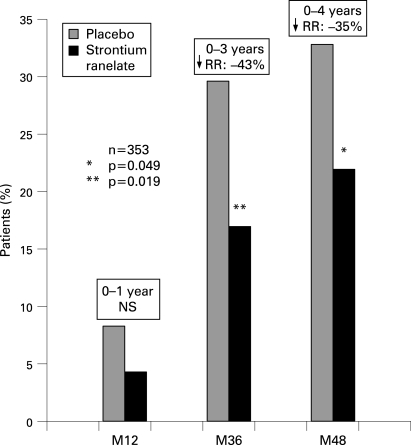
Incidence of vertebral fractures over the study period (1 (M12), 3 (M36) and 4 (M48) years data). RR, relative risk.

Over 3 years, treatment with strontium ranelate significantly reduced the risk of vertebral fracture by 43% (RR 0.57; 95% CI 0.36 to 0.92, p = 0.019), with an incidence of vertebral fractures of 16.9% in the strontium ranelate group versus 29.6% in the placebo group. This efficacy in reducing the risk of vertebral fractures was sustained over 4 years of treatment with strontium ranelate, with a reduction by 35% (RR 0.65, 95% CI 0.42 to 0.99, p = 0.049) and an incidence of vertebral fractures of 21.6% in the strontium ranelate group versus 32.8% in the placebo group. There was a trend towards a reduction in the risk of vertebral fracture over the first year, which was not statistically significant (fig 1). A significant effect of strontium ranelate compared with placebo was also observed regarding symptomatic vertebral fractures (defined as radiological fractures plus concomitant back pain or a decrease in body height by at least 1 cm) with a 54% reduction in the risk of symptomatic vertebral fracture over 3 years (RR 0.46; 95% CI 0.22 to 0.97, p = 0.033), sustained over 4 years with a 52% reduction (RR 0.48; 95% CI 0.24 to 0.95, p = 0.030). There was no difference in the incidence of non-vertebral fractures between groups over 4 years (14.5% and 14.6% in the treated and placebo groups, respectively).

**Figure 2:**
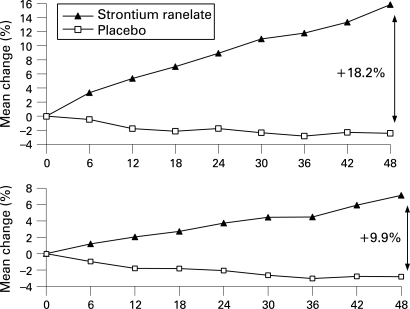
Changes in spine and hip bone mineral density over 4 years in postmenopausal women with osteoporosis aged 50–65 years receiving either placebo or strontium ranelate.

As shown in fig 2, the BMD mean change from baseline over 3 years at the spine and hip sites was 11.8% and 4.5%, respectively, for the strontium ranelate-treated group, and −2.8% and −3.0% at these two sites in the placebo group. Over 4 years, BMD increased further in the strontium ranelate group, with a mean change of 15.8% and 7.1% at the spine and hip, whereas it remained lower than baseline values in the placebo group, with mean changes of −2.4% and −2.8% at these two sites (significant difference between groups, p<0.001 at both sites).

Over the 4-year follow-up, clinical, serious, drug-related adverse effects were similar in the placebo and treated groups (no clinical significance between groups regarding the incidence of nausea, diarrhoea, headache, dermatitis, eczema, venous thromboembolism events) and the overall safety profile was very similar to that already described for the whole SOTI study population over 3 and 4 years. No case of pulmonary embolism or hypersensitivity reaction was observed in this study population.

## DISCUSSION

This study shows that strontium ranelate (2 g/day) decreases the risk of vertebral fractures over 4 years in women aged 50–65 years with severe osteoporosis.

The fracture incidence increases with age, but young postmenopausal women experience fractures. In the National Osteoporosis Risk Assessment (NORA) study, 37% of the fractures occurred in women 50–64 years of age, and this population of young postmenopausal women accounted for one-fifth of the hip fractures that occurred within 1 year of baseline.^9^ A prevalent fracture may be a much more severe predictor of subsequent fractures than other well-known risk factors when considering younger populations at risk due to the fact that the prevalence of fractures largely increases with age. Such a prevalent fracture is more common in communities aged 70 or 80 years than in younger populations, giving less weight in terms of risk to this factor in the elderly.^10^ In addition, it has been shown in a general semi-urban population that the presence of vertebral fractures led to a decrease in the expected remaining life years, the decrease being greater in the younger than in the older age groups.^11^ This highlights the importance of diagnosing and treating appropriately as soon as possible this youngest postmenopausal population at high further risk.

In the present study, the incidence of vertebral and non-vertebral fractures over 4 years in the placebo group was high (32.8% and 14.6%, respectively). This demonstrates in this population aged less than 65 years that the presence of prevalent fractures leads to a dramatic increase in the overall fracture risk, not limited to vertebral fractures. Over the first year, the incidence of vertebral fractures was 8.3%, which reinforces the indication of a prompt and effective treatment in this population.

As strontium is a heavier element than calcium, its presence in bone could lead to an overestimation of BMD measurement. However, a strong association has been demonstrated for strontium ranelate-treated patients between the increase in total hip and/or femoral neck BMD and the subsequent decrease in fracture incidence.^12^

Few studies are available with other antiosteoporotic drugs in women aged 50–65 years. The Women’s Health Initiative showed that treatment with oestrogens alone, or oestrogen plus progestin, reduces the incidence of fractures compared with control,^13^ but the study was conducted in healthy postmenopausal women. Young age was not a significant factor in affecting the efficacy of raloxifene, in a population of postmenopausal women with a prevalence of 37% of vertebral fractures.^14^ Several studies have shown the absence of effect of age on response to antiosteoporotic treatment, but this point was assessed either in patients older than 65 years^15^ or in a population with a mean age of 70 years.^16^

Our study indicates a significant efficacy of strontium ranelate in reducing the risk of subsequent vertebral fractures in young postmenopausal women with severe osteoporosis. These data together with previous reports confirm the efficacy of this antiosteoporotic drug at all ages.
